# The dark side of histones: genomic organization and role of oncohistones in cancer

**DOI:** 10.1186/s13148-021-01057-x

**Published:** 2021-04-07

**Authors:** Stefano Amatori, Simona Tavolaro, Stefano Gambardella, Mirco Fanelli

**Affiliations:** 1 Department of Biomolecular Sciences, Molecular Pathology Laboratory “PaoLa”, University of Urbino Carlo Bo, Via Arco d’Augusto 2, 61032 Fano, PU Italy; 2 Fredis Associazione, Via Edoardo Jenner 30, 00151 Rome, Italy; 3 IRCCS Neuromed, Via Atinense 18, 86077 Pozzilli, IS Italy

**Keywords:** Histones, Histone genes, Histone mutations, Cancer, Oncohistones

## Abstract

**Background:**

The oncogenic role of histone mutations is one of the most relevant discovery in cancer epigenetics. Recurrent mutations targeting histone genes have been described in pediatric brain tumors, chondroblastoma, giant cell tumor of bone and other tumor types. The demonstration that mutant histones can be oncogenic and drive the tumorigenesis in pediatric tumors, led to the coining of the term “oncohistones.” The first identified histone mutations were localized at or near residues normally targeted by post-translational modifications (PTMs) in the histone *N*-terminal tails and suggested a possible interference with histone PTMs regulation and reading.

**Main body:**

In this review, we describe the peculiar organization of the multiple genes that encode histone proteins, and the latter advances in both the identification and the biological role of histone mutations in cancer. Recent works show that recurrent somatic mutations target both *N*-terminal tails and globular histone fold domain in diverse tumor types. Oncohistones are often dominant-negative and occur at higher frequencies in tumors affecting children and adolescents. Notably, in many cases the mutations target selectively only some of the genes coding the same histone protein and are frequently associated with specific tumor types or, as documented for histone variant H3.3 in pediatric glioma, with peculiar tumors arising from specific anatomic locations.

**Conclusion:**

The overview of the most recent advances suggests that the oncogenic potential of histone mutations can be exerted, together with the alteration of histone PTMs, through the destabilization of nucleosome and DNA–nucleosome interactions, as well as through the disruption of higher-order chromatin structure. However, further studies are necessary to fully elucidate the mechanism of action of oncohistones, as well as to evaluate their possible application to cancer classification, prognosis and to the identification of new therapies.

## Background

Chromatin structure plays a fundamental role in the regulation of crucial cellular processes including replication, maintenance of genomic stability and regulation of gene expression, through which it controls functions such as cell cycle, DNA repair and cell fate. The eukaryotic chromatin is confined to a specialized cellular compartment, the nucleus and consists of a nucleoprotein complex of genomic DNA, histones and non-histone proteins with an advanced level of organization. The basic units of chromatin are nucleosomes, which are composed of 147 base pairs of DNA wrapped ∼ 1.7 turns around a histone octamer core. The octamer contains four heterodimers of the core histone proteins H2A, H2B, H3, and H4. A fifth histone, histone H1, binds to internucleosomal DNA (named “linker DNA”) to stabilize higher-order chromatin structures. The regulation of chromatin function is mainly based on histone post-translational modifications (PTMs), which consist of enzyme-mediated chemical modifications of specific histone residues, in particular at the level of the *N*-terminal tail, known for many years as epigenetic modifications. These modifications, combined with the activity of different ATP-dependent chromatin remodelers, control the chromatin state and thus play a major role in regulating gene expression, making chromatin accessible or not to transcriptional regulatory complexes [1].

The canonical histones (H3, H4, H2A, H2B) are the most abundant in nucleosomes and are synthesized and incorporated in a replication-dependent manner. In humans, exception made for histone H4, several variants of “canonical” histones have been described with significant differences in primary sequence [2, 3]. Histone H3 variants have attracted special attention in last years, and the incorporation of histone variants has shown to provide a further level of gene expression regulation by conferring specific states to chromatin through their dynamic exchange with canonical histones [4–9].

In the light of this evidence, it is not surprising that chromatin deregulation was found to play a pivotal role in many human diseases, including cancer. The function of many histone-modifying enzymes and chromatin remodeling complexes is often compromised in cancer, and these alterations are considered key mechanisms in tumor development and progression [10, 11]. Similarly, aberrant expression and/or incorporation of histone variants has been linked to cancer and, in particular, to more aggressive cancer phenotypes [12, 13]. In recent years, histone mutations have been discovered in several cancer types and many proofs to sustain their role in tumor transformation have been accumulated. Peculiar features of these mutants led to the coining of the term “oncohistones” and to the belief that they may function as drivers of tumorigenesis [14, 15].

In this review, we aim to examine the genomic organization of histone-coding genes and their mutations and to shed light on recent advances in understanding how these mutations can lead to chromatin aberrations and, eventually, contribute to cancer development.

## The genomic organization of histones in eukaryotes

### Canonical histones

**Fig. 1:**
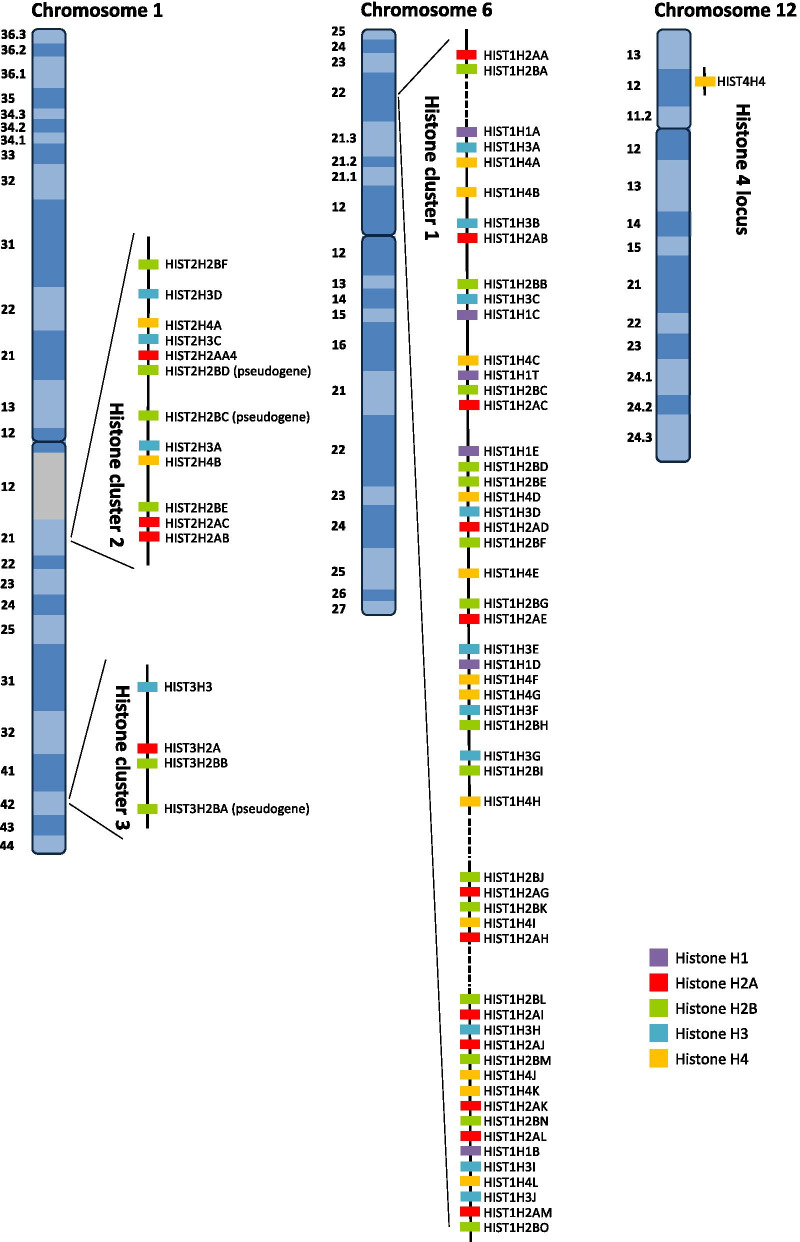
Genomic organization of canonical histone genes in humans. The distribution of canonical histone genes within the histone cluster 1, histone cluster 2, histone cluster 3 and histone 4 locus on human chromosomes is shown. The genes belonging to the histone H1 family are indicated in purple, the genes of the H2A family in red, the genes of the H2B family in green, the genes of the H3 family in blue and the genes of the H4 family in yellow, respectively

Histones represent about half of the eukaryotic chromosome mass and are a family of basic proteins among the most abundant in eukaryotic cells, as well as one of the most evolutionarily conserved. As already stated, the majority of histones present in eukaryotic cells are part of canonical histones [3]. These proteins are encoded by multiple gene copies that are organized in clusters on different chromosomes (Fig. 1) [16]. A total of 73 histone genes have been identified in humans, including 16 genes for histone H2A (Table 1), 22 genes for histone H2B (Table 2), 14 genes for histone H3 (Table 3), 15 genes for histone H4 (Table 4) and 6 genes for histone H1, along with the genes encoding for the testis-specific variants TSH2B.1, H3.1t and H1t (Table 5) [17]. Chromosome 6 (HIST1 locus) contains the major cluster of these genes (55 genes, ∼ 80% of the total) [3], while the other histones are located in two smaller clusters, the histone clusters 2 and 3 (HIST2 and HIST3 loci), on chromosome 1 (Fig. 1). At variance, the HIST4H4 gene, encoding for the H4 core histone, is present on chromosome 12 in histone cluster 4 (HIST4 locus—Fig. 1) [3, 17]. In view of the important role of replication-coupled histones in the cell, this redundancy probably ensures a continuous source of core histone proteins during cellular replication. Moreover, the organization in cluster of the histone genes seems to indicate an evolutionary selection that favored an optimal mRNA processing during the biosynthesis of these proteins. Of note, as shown by a comparison of core histone sequences, these genes are not completely identical. In fact, core histones present different subtypes: in particular, 11 isoforms for H2A (Table 1), 14 for H2B (including the testis-specific variant TSH2B.1—Table 2), 3 for H3 (including the testis-specific variant H3.1t—Table 3) and 2 for H4 (Table 4), respectively (Table 1). With the exception of HIST1H2BA and HIST1H4G, these isoforms display more than 90% of similarity with the corresponding core histones, therefore encoding for proteins with minimal structural changes [17]. This heterogeneity, albeit marginal, probably may have an effect on nucleosome structure.

**Table 1:** Human canonical and non-canonical core histones of the H2A family with their chromosomal location, expression and function

	Gene name*	Histone protein codified	Gene chromosomal location	Expression	Function	References
*Canonical H2A core histones*						
1	HIST1H2AA	H2A type 1-A	6p22.2 (in histone cluster 1)	Replication-dependent	Core component of nucleosome	[18]
2	HIST1H2AB	H2A type 1-B/E	6p22.2 (in histone cluster 1)	Replication-dependent	Core component of nucleosome	[18]
3	HIST1H2AE		6p22.2 (in histone cluster 1)			
4	HIST1H2AC	H2A type 1-C	6p22.2 (in histone cluster 1)	Replication-dependent	Core component of nucleosome	[18]
5	HIST1H2AD	H2A type 1-D	6p22.2 (in histone cluster 1)	Replication-dependent	Core component of nucleosome	[18]
6	HIST1H2AH	H2A type 1-H	6p22.1 (in histone cluster 1)	Replication-dependent	Core component of nucleosome	[18]
7	HIST1H2AJ	H2A type 1-J	6p22.1 (in histone cluster 1)	Replication-dependent	Core component of nucleosome	[18]
8	HIST1H2AG	H2A type 1	6p22.1 (in histone cluster 1)	Replication-dependent	Core component of nucleosome	[18]
9	HIST1H2AI		6p22.1 (in histone cluster 1)			
10	HIST1H2AK		6p22.1 (in histone cluster 1)			
11	HIST1H2AL		6p22.1 (in histone cluster 1)			
12	HIST1H2AM		6p22.1 (in histone cluster 1)			
13	HIST2H2AA	H2A type 2-A	1q21.2 (in histone cluster 2)	Replication-dependent	Core component of nucleosome	[18]
14	HIST2H2AB	H2A type 2-B	1q21.2 (in histone cluster 2)	Replication-dependent	Core component of nucleosome	[18]
15	HIST2H2AC	H2A type 2-C	1q21.2 (in histone cluster 2)	Replication-dependent	Core component of nucleosome	[18]
16	HIST3H2A	H2A type 3	1q42.13 (in histone cluster 3)	Replication-dependent	Core component of nucleosome	[18]
*Non-canonical H2A core histones*						
1	H2AX	H2A.X	11q23.3	Replication-independent	Required for cell cycle arrest and DNA repair after DNA damage	[19–22]
2	H2AZ1	H2A.Z	4q23	Replication-independent	Involvement in the formation of constitutive heterochromatin and in gene expression regulation	[23, 24]
3	H2AZ2	H2A.Z.2.1	7p13	Replication-independent (enriched in brain)	Involvement in the binding of regulatory complexes	[25]
		H2A.Z.2.2			Destabilization of nucleosome	[26]
4	H2AB1, H2AB2 and H2AB3	H2A.Bbd type1, H2A.Bbd type2 and H2A.Bbd type3	Xq28	Replication-independent	Required for mRNA processing and association with active transcription and replication	[27, 28]
5	MACROH2A1	macroH2A1.1	5q31.1	Replication-independent	Involvement in ADP-ribose-mediated chromatin modulation and increase in the expression of genes involved in redox metabolism (such as SOD3)	[29, 30]
		macroH2A1.2		Replication-independent	Repression of SOD3 gene expression	[30]
6	MACROH2A2	macroH2A2	10q22.1	Replication-independent	Involvement in stable X chromosome inactivation	[31]

*The gene names of canonical histones are composed by a first part referring to their histone cluster, followed by the type of histone and, finally, by a letter, designated in alphabetical order for each histone type, based on the distance from the telomere

**Table 2:** Human canonical and non-canonical core histones of the H2B family with their chromosomal location, expression and function

	Gene name*	Histone protein codified	Gene chromosomal location	Expression	Function	References
*Canonical H2B core histones*						
1	HIST1H2BA (or TSH2B)	H2B type 1-A (or TSH2B.1)^§^	6p22.2 (in histone cluster 1)	Replication-dependent and tissue-specific (testis/sperm-specific)	Not determined	[32]
2	HIST1H2BB	H2B type 1-B	6p22.2 (in histone cluster 1)	Replication-dependent	Core component of nucleosome	[18]
3	HIST1H2BC	H2B type 1-C/E/F/G/I	6p22.2 (in histone cluster 1)	Replication-dependent	Core component of nucleosome	[18]
4	HIST1H2BE					
5	HIST1H2BF					
6	HIST1H2BG					
7	HIST1H2BI					
8	HIST1H2BD	H2B type 1-D	6p22.2 (in histone cluster 1)	Replication-dependent	Core component of nucleosome	[18]
9	HIST1H2BH	H2B type 1-H	6p22.2 (in histone cluster 1)	Replication-dependent	Core component of nucleosome	[18]
10	HIST1H2BJ	H2B type 1-J	6p22.1 (in histone cluster 1)	Replication-dependent	Core component of nucleosome	[18]
11	HIST1H2BK	H2B type 1-K	6p22.1 (in histone cluster 1)	Replication-dependent	Core component of nucleosome	[18]
12	HIST1H2BL	H2B type 1-L	6p22.1 (in histone cluster 1)	Replication-dependent	Core component of nucleosome	[18]
13	HIST1H2BM	H2B type 1-M	6p22.1 (in histone cluster 1)	Replication-dependent	Core component of nucleosome	[18]
14	HIST1H2BN	H2B type 1-N	6p22.1 (in histone cluster 1)	Replication-dependent	Core component of nucleosome	[18]
15	HIST1H2BO	H2B type 1-O	6p22.1 (in histone cluster 1)	Replication-dependent	Core component of nucleosome	[18]
16	HIST2H2BB (pseudogene)		1q21.1 (in histone cluster 2)			
17	HIST2H2BC (pseudogene)		1q21.2 (in histone cluster 2)			
18	HIST2H2BD (pseudogene)		1q21.2 (in histone cluster 2)			
19	HIST2H2BE	H2B type 2-E	1q21.2 (in histone cluster 2)	Replication-dependent	Core component of nucleosome	[18]
20	HIST2H2BF	H2B type 2-F	1q21.2 (in histone cluster 2)	Replication-dependent	Core component of nucleosome	[18]
21	HIST3H2BA (pseudogene)		1q42.13 (in histone cluster 3)			
22	HIST3H2BB	H2B type 3-B	1q42.13 (in histone cluster 3)	Replication-dependent	Core component of nucleosome	[18]
*Non-canonical H2B core histones*						
1	H2BW1	H2B.W	Xq22.2	Replication-independent and tissue-specific (testis/sperm-specific)	Involvement in telomere function. Unlike conventional H2B, H2B.W1 does not recruit chromosome condensation factors and does not participate in the assembly of mitotic chromosomes	[33]

*The gene names of canonical histones are composed by a first part referring to their histone cluster, followed by the type of histone and, finally, by a letter, designated in alphabetical order for each histone type, based on the distance from the telomere

^§^These isoforms are testis-specific variants

**Table 3:** Human canonical and non-canonical core histones of the H3 family with their chromosomal location, expression and function

	Gene name*	Histone protein codified	Gene chromosomal location	Expression	Function	References
*Canonical H3 core histones*						
1	HIST1H3A	H3.1	6p22.2 (in histone cluster 1)	Replication-dependent	Core component of nucleosome	[18]
2	HIST1H3B		6p22.2 (in histone cluster 1)	Replication-dependent	Core component of nucleosome	[18]
3	HIST1H3C		6p22.2 (in histone cluster 1)	Replication-dependent	Core component of nucleosome	[18]
4	HIST1H3D		6p22.2 (in histone cluster 1)	Replication-dependent	Core component of nucleosome	[18]
5	HIST1H3E		6p22.2 (in histone cluster 1)	Replication-dependent	Core component of nucleosome	[18]
6	HIST1H3F		6p22.2 (in histone cluster 1)	Replication-dependent	Core component of nucleosome	[18]
7	HIST1H3G		6p22.2 (in histone cluster 1)	Replication-dependent	Core component of nucleosome	[18]
8	HIST1H3H		6p22.1 (in histone cluster 1)	Replication-dependent	Core component of nucleosome	[18]
9	HIST1H3I		6p22.1 (in histone cluster 1)	Replication-dependent	Core component of nucleosome	[18]
10	HIST1H3J		6p22.1 (in histone cluster 1)	Replication-dependent	Core component of nucleosome	[18]
11	HIST2H3A	H3.2	1q21.2 (in histone cluster 2)	Replication-dependent	Core component of nucleosome	[18]
12	HIST2H3C		1q21.2 (in histone cluster 2)	Replication-dependent	Core component of nucleosome	[18]
13	HIST2H3D		1q21.2 (in histone cluster 2)	Replication-dependent	Core component of nucleosome	[18]
14	HIST3H3 (or H3-4)	H3.1t (or H3.4)^§^	1q42.13 (in histone cluster 3)	Replication-dependent and tissue-specific (testis-specific)	Core component of nucleosome	[18, 34]
*Non-canonical H3 core histones*						
1	H3-3A and H3-3B	H3.3	1q42.12 and 17q25.1	Replication-independent	Induction of gene activation and involvement in gene expression regulation	[35, 36]
2	CENPA	CENP-A	2p23.3	Replication-independent	Required for recruitment and assembly of kinetochore proteins and progression through mitosis, chromosome segregation and cytokinesis	[37–40]
3	H3F3C (or H3-5)	H3.3C (or H3.5)	12p11.21	Replication-independent (specifically in seminiferous tubules of testis)	Colocalization with euchromatin and association with actively transcribed genes	[41]
4	H3Y2	H3.X (or H3.Y2)	5p15.1	Replication-independent	Not yet clarified	[42]
5	H3Y1	H3.Y (or H3.Y1)	5p15.1	Replication-independent	Involvement in the regulation of cellular responses to stress stimuli and regulation of transcription	[42, 43]

*The gene names of canonical histones are composed by a first part referring to their histone cluster, followed by the type of histone and, finally, by a letter, designated in alphabetical order for each histone type, based on the distance from the telomere

^§^These isoforms are testis-specific variants

**Table 4:** Human canonical core histones of the H4 family with their chromosomal location, expression and function

	Gene name*	Histone protein codified	Gene chromosomal location	Expression	Function	References
*Canonical H4 core histones*						
1	HIST1H4A	H4	6p22.2 (in histone cluster 1)	Replication-dependent	Core component of nucleosome	[18]
2	HIST1H4B		6p22.2 (in histone cluster 1)	Replication-dependent	Core component of nucleosome	[18]
3	HIST1H4C		6p22.2 (in histone cluster 1)	Replication-dependent	Core component of nucleosome	[18]
4	HIST1H4D		6p22.2 (in histone cluster 1)	Replication-dependent	Core component of nucleosome	[18]
5	HIST1H4E		6p22.2 (in histone cluster 1)	Replication-dependent	Core component of nucleosome	[18]
6	HIST1H4F		6p22.2 (in histone cluster 1)	Replication-dependent	Core component of nucleosome	[18]
7	HIST1H4H		6p22.2 (in histone cluster 1)	Replication-dependent	Core component of nucleosome	[18]
8	HIST1H4I		6p22.1 (in histone cluster 1)	Replication-dependent	Core component of nucleosome	[18]
9	HIST1H4J		6p22.1 (in histone cluster 1)	Replication-dependent	Core component of nucleosome	[18]
10	HIST1H4K		6p22.1 (in histone cluster 1)	Replication-dependent	Core component of nucleosome	[18]
11	HIST1H4L		6p22.1 (in histone cluster 1)	Replication-dependent	Core component of nucleosome	[18]
12	HIST2H4A		1q21.2 (in histone cluster 2)	Replication-dependent	Core component of nucleosome	[18]
13	HIST2H4B		1q21.2 (in histone cluster 2)	Replication-dependent	Core component of nucleosome	[18]
14	HIST4H4		12p12.3 (in histone cluster 4)	Replication-dependent	Core component of nucleosome	[18]
15	HIST1H4G	H4-like protein type G	6p22.2 (in histone cluster 1)	Replication-dependent	Core component of nucleosome	[18]

*The gene names of canonical histones are composed by a first part referring to their histone cluster, followed by the type of histone and, finally, by a letter, designated in alphabetical order for each histone type, based on the distance from the telomere

**Table 5:** Human canonical and non-canonical linker histones of the H1 family with their chromosomal location, expression and function

	Gene name*		Histone protein codified	Gene chromosomal location	Expression	Function	References
	*Canonical H1 linker histones*						
1	HIST1H1A		H1.1	6p22.2 (in histone cluster 1)	Replication-dependent	Linker histone essential for the condensation of nucleosome chains into higher-order structured fibers	[18, 44]
2	HIST1H1C		H1.2	6p22.2 (in histone cluster 1)	Replication-dependent	Linker histone essential for the condensation of nucleosome chains into higher-order structured fibers	[18, 44]
3	HIST1H1D		H1.3	6p22.2 (in histone cluster 1)	Replication-dependent	Linker histone essential for the condensation of nucleosome chains into higher-order structured fibers	[18, 44]
4	HIST1H1E		H1.4	6p22.2 (in histone cluster 1)	Replication-dependent	Linker histone essential for the condensation of nucleosome chains into higher-order structured fibers	[18, 44]
5	HIST1H1B		H1.5	6p22.1 (in histone cluster 1)	Replication-dependent	Linker histone essential for the condensation of nucleosome chains into higher-order structured fibers	[18, 44]
6	HIST1H1T (or H1-6)		H1t (or H1.6)^§^	6p22.2 (in histone cluster 1)	Replication-dependent and tissue-specific (testis-specific)	Formation of more relaxed chromatin	[45]
*Non-canonical linker histone H1*							
1	H1FNT (or H1-7)	H1t2 (or H1.7)		12q13.11	Replication-independent and tissue-specific (testis-specific)	Required for spermatid elongation and DNA condensation during spermiogenesis (essential for normal spermatogenesis and male fertility)	[46]
2	H1FOO (or H1-8)	H1oo (or H1.8)		3q22.1	Replication-independent and tissue-specific (oocyte-specific)	Regulation of gene expression during oogenesis and early embryogenesis	[47]
3	HILS1 (or H1-9)	H1.9		17q21.33	Replication-independent and tissue-specific (testis-specific)	Involvement in chromatin remodeling during mammalian spermiogenesis	[48]
4	H1FX (or H1-10)	H1x (or H1.10)		3q21.3	Replication-independent	Required for mitotic progression	[49]
5	H1-0	H1.0		22q13.1	Replication-independent	Required for the terminal stages of differentiation	[50, 51]

*The gene names of canonical histones are composed by a first part referring to their histone cluster, followed by the type of histone and, finally, by a letter, designated in alphabetical order for each histone type, based on the distance from the telomere

^§^These isoforms are testis-specific variants

Compared with the other canonical histones, the 5 family members of the linker histone H1 (H1.1, H1.2, H1.3, H1.4, and H1.5) show the highest sequence variability among the organisms. However, also this linker histone is encoded in a replication-dependent manner and organized in the histone gene cluster along with the core histone genes [16, 52]. In particular, all of the genes of the histone H1 family are located in histone cluster 1 at chromosome 6, including the testis-specific variant H1t (Fig. 1, Table 5).

### Non-canonical histones

Beyond the canonical histones, different histone variants are expressed in eukaryotic cells: the “non-canonical” histones, characterized by significant differences in primary sequence compared to canonical histones. Unlike the canonical histones and except for TSH2B.1, H3.1t and H1t histone variants, that shown a DNA replication-dependent expression, the other variants are included in the nucleosomes in a DNA replication-independent way and are expressed throughout the cell cycle and/or in a tissue-specific manner. More specifically, while the newly assembled nucleosomes containing the canonical histones are included in the gaps between old nucleosomes, histones variants replace existing nucleosomal subunits locally. As discussed below, this feature ensures a unique genomic spatial distribution that, in turn, is strictly regulated by a complex machinery of specific chaperones and ATP-dependent chromatin remodelers [53–55]. Another difference between canonical and non-canonical subtypes is that the histone variants are encoded by individual, rather than multiple genes, that are usually located on different chromosomes than those of canonical histones [3]. Moreover, the genes codifying the canonical histones lack introns and contain a conserved palindromic sequence of termination which replaces the poly(A) tail and form a specific stem-loop structure involved in the recognition by the stem-loop-binding proteins at 3ʹ during mRNA transcription [16, 56]. Contrariwise, non-canonical gene variants often contain introns, thus generating different alternative isoforms that may contribute to produce nucleosome diversity as well as to regulate gene expression, as discussed below [3, 57]. Additionally, mRNAs of non-canonical histones present poly(A) tails, like the typical eukaryotic transcripts [3, 58]. To date, more than 50 non-canonical histones are known in mammals [2].

Human somatic cells express several variants of the core histone proteins [59]. In particular, the majority of these variants have been identified for H2A and H3 (Table 1 and Table 3) [60]; in fact, Buschbeck et al. [17] reported eight variants of H2A, including H2A.X, H2A.Z.1, H2A.Z.2.1, H2A.Z.2.2, H2A.B (also known as H2A.Bbd), macroH2A1.1, macroH2A1.2 and macroH2A2 (Table 1), and six variants of histone H3, namely H3.3, CENP-A (histone H3 like centromeric protein A), H3.1T (also known as H3.4), H3.3C (also known as H3.5), H3.X (also known as H3.Y2) and H3.Y (also known as H3.Y1—Table 3). On the contrary, only two isoforms of histone H2B, H2B.W and TSH2B, are expressed in testis (Table 2) [3], while no variant has yet been discovered for H4 (Table 4) [59]. Although some of these variants display minimal sequence changes (such as for H3.3), compared to the respective canonical histones, other ones show substantial differences that lead to important structural modifications (as for macroH2A and CENP-A) [61]. Moreover, it is worth mentioning that H2A variants are the only subtypes that cause alterations belonging to the *N*-terminal of core histones rather than to the *C*-terminal, as occurs in the other non-canonical proteins [2].

Concerning the linker histones, 11 variants have been found in mice and humans (Table 5) [8, 62]. In humans, besides the five somatic, replication-dependent, family members H1.1–H1.5, four variants exhibit a germ cell-specific expression, more specifically, H1oo (also known as H1.8) in oocytes and H1t, H1t2 (also known as H1.6 and H1.7, respectively) and H1.9 in spermatids or spermatocytes [46, 63, 64]. Besides, other two replication-independent subtypes have been found, i.e., H1.0, that seems to replace the H1 somatic variants in differentiated cells, and H1x, also known as H1.10 (Table 5) [50, 65].

Despite the large number of non-canonical histones, these proteins are evolutionarily conserved among the different species, suggesting an important role played by these variants in eukaryotes. In line with these observations, it has been shown that non-canonical subtypes not only replace the missing histones, thus acting like their canonical counterparts as scaffold proteins that condense eukaryotic genomes, but also contribute to changing chromatin composition [60]. The incorporation of histone variants can have a direct effect on the structure and stability of nucleosomes also modifying, in specific cases, the accessibility of DNA and, as a consequence, the regulation of crucial cellular processes, such as transcription, replication and DNA repair and recombination [3, 8, 60, 66]. This explains why the nucleosomes containing the histone variants are not equally distributed along the chromatin but rather according to specific positions [3]. For example, H2A.Z, that is the most conserved H2A variant across species, is found enriched at the 5′ of many genes, as well as, macroH2A accumulates on the inactive X chromosome [67, 68]. Moreover, when the histone variant H2A.Bbd replaces another protein of the core, given the different conformation of the C-terminal of this isoform, the stability of nucleosome is reduced making chromatin less condensed and, in turn, more available for transcription [69, 70]. On the other hand, while the H3 variant H3.3 is enriched at promoters and transcriptionally active sites [71], CENP-A is incorporated into specific nucleosomes to form a functional centromere, as demonstrated by several experiments based on depletion of CENP-A in human cells, making the role of this protein fundamental for kinetochore assembly and integrity [37, 72].

Since the core histone proteins are subject to a large number of covalent PTMs at their N-terminal tails, the recruitment of histone variants in nucleosomes may also indirectly influence the epigenetic cellular profile altering, at the same time, the “open” or “closed” state of chromatin as well as the gene expression regulation [3, 60, 73–75]. Considering that the expression of non-canonical variants is regulated in a time- and tissue-dependent manner, this dynamic process is operational also during embryonic development and cell differentiation [3, 53].

## Oncohistones

Histone alterations are known to play a pivotal role in tumorigenesis. Aberrant distribution of histone PTMs in cancer has been indeed largely documented and linked to the malfunction of the enzymatic machinery that regulates their writing, erasing and reading [10], the deregulation of chromatin remodeling complexes [11], as well as the aberrant expression and/or incorporation of specific histone variants [12, 13]. Starting from 2012, somatic mutations have been discovered to be highly frequent in several histone-coding genes [76, 77]. Similarly to mutations of chromatin-associated or regulatory protein, the importance of histone mutations in cancer is highlighted by their frequency in tumors affecting children and adolescents. Interestingly, in many cases histone mutants exert dominant effects regardless of the presence of several not-mutated copies of their genes and with the mutant protein constituting only a small fraction compared to the other histone proteins of the same family [14].

A great contribution to the advances in our knowledge of the distribution of histone mutations in cancer comes from two recent works that catalogued and characterized the landscape of missense histone mutations in thousands of patients across several tumor types [78, 79]. These works were based on data from cBioPortal to shed further light on specific attributes of histone mutations causing recurrent amino acidic transitions associated with specific tumor types or, as already documented in previous studies on histone variant H3.3 in pediatric glioma, with peculiar tumors arising from specific anatomic locations [78–81].

Due to the numerous histone mutations identified in cancer to date, we will describe the main mutations in core histones and their prominent variants, focusing on the most frequent and functionally characterized ones, and the one found to be tightly associated with specific tumor types.

## Histone H3 mutations

**Fig. 2:**
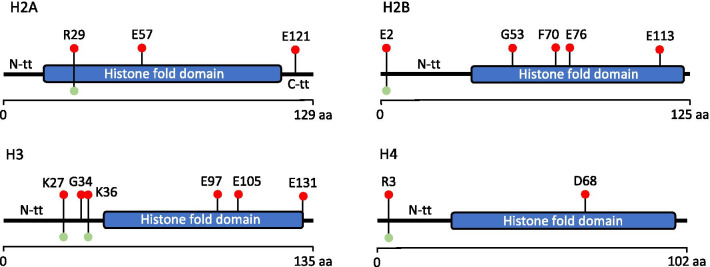
Localization of cancer-associated histone mutations. The most relevant somatic missense histone mutations for each core histone family are shown (red circles). Mutations were considered relevant depending on their recurrence in cross-cancer mutation summaries using cBioPortal and/or on the basis of the existence of studies that investigated their functional role. Globular domains are indicated by blue bars; sites of known PTMs are indicated by green circles. N-tt, N-terminal tail; C-tt, C-terminal tail

Histone H3 was the first histone found to be frequently mutated in cancer. Initially discovered by two separate groups in 2012, the association between histone H3 **K27**M mutation and pediatric glioblastoma (pGBM) was confirmed by many other studies [76, 77, 80–83]. In the last few years, the list of cancers known to carry mutations in H3 has progressively lengthened to include chondroblastoma, chondrosarcoma, osteosarcoma, head and neck squamous cell carcinoma, pediatric soft tissue sarcoma, bladder cancer, melanoma and acute myeloid leukemia [78, 79, 84–89]. Recent studies confirmed that the most frequently mutated histone in cancer is histone H3, suggesting a major role in the regulation of gene expression and/or chromatin assembly [78, 79]. Although the most studied histone H3 mutations, such as **K27**M, **K36**M and **G34** mutants, localize in the N-terminal domain and are thought to play their oncogenic role by perturbing the pattern of histone PTMs, there are also several residues in the globular domain, such as **E97**, **E105** and **R131** that have been found mutated at similar or even higher rates (Fig. 2, Table 6) [78, 79, 90]. Differently from what was believed few years ago indeed, cancer relevant histone mutations are not limited to known PTMs-targeted histone tail or nearby residues but are also distributed along the whole gene body. Frequent mutations map in regions important for nucleosome integrity and/or DNA–nucleosome interactions, suggesting that the loss of nucleosome structure and the perturbation of higher-order chromatin may be a major mechanism of the oncohistone-mediated carcinogenesis [78, 79].

**Table 6:** Main histone mutations in cancer

Histone	Mutations	Preferential genes	Histone domain	Proposed mechanism	Biological evidence	Primary sites of cancer	References
H2A	E121Q/K/D	Mainly Hist1 cluster	C-TT	Reduced interaction of H2A-H2B dimer with H3-H4 dimer and H2A with linker DNA	None	None	[78, 79]
	R29Q	None	HFD	Interference with residue methylation	None	None	[78, 79]
	E57Q	HIST1H2AB	HFD	Interference with the structural integrity of the H2A fold	Increased expression of EMT markers (in vitro); increased migratory; and invasive phenotype (in vitro)	Carcinomas of the female tract	[91]
H2B	E76K	Mainly Hist1 cluster	HFD	Interference with H2A-H2B interaction; distortion of the interface between H2B and H4	Enhanced colony formation; cellular proliferation (in vitro); cooperation with mutant PIK3CA to promote colony formation (in vitro)	Bladder cancer, head and neck cancer	[79, 90]
	E113K/Q	None	HFD	Interference with acidic patch at H2A-H2B dimer surface	None	None	[78, 79]
	F70L	Mainly Hist2 cluster	HFD	Distortion of H2B-H4 interface (adjacent residue E71 is also frequently mutated)	None	None	[78, 79]
	E2Q/K	None	N-TT	Interference with residue ADP-ribosylation	None	None	[78, 79]
	G53D	Mainly Hist2 cluster	H HFD	Disruption of H2B-DNA interaction	Increased gap closure ability and transwell migration of cells (in vitro)	Pancreatic ductal adenocarcinoma	[79, 92]
	S37many	None	HFD	Interference with residue phosphorylation	None	Follicular lymphoma	[93]
H3	K27M	H3F3A (H3.3)	N-TT	Interference with residue methylation (dominant-negative)	Tumor growth and differentiation (in vitro and in vivo); associated with more aggressive tumor phenotype (in patients)	Diffuse intrinsic pontine glioma (pediatric)	[76–79, 83, 85, 94–108]
	K36M	H3F3B (H3.3)	N-TT	Interference with residue methylation (dominant-negative)	Tumor development (in vivo); enhanced colony formation and less sensitivity to apoptotic stimuli (in vitro)	Chondroblastoma (children and young adults)	[78, 84, 88, 109, 110]
	G34R/V	H3F3A (H3.3)	N-TT	Interference with K36 and K27 methylation (dominant-negative)	None	Cortical pediatric high-grade glioma (young adults)	[76, 94]
	G34W/L	H3F3A (H3.3)	N-TT	Interference with K36 and K27 methylation (dominant-negative); interference with DNA methylation and histone modifications at heterochromatic and bivalent regions	Enhanced colony formation, infiltration and proliferation (G34W—in vitro and in vivo); reduced differentiation of GCTB stromal cells (primary cells)	Giant cell tumors of bone	[84, 111]
	E97K	H3.1 genes	HFD	Interference with nucleosome structure	Enhanced colony formation (in vitro)	None	[78, 79, 90]
	E105K/V	H3B (slight preference)	HFD	Interference with nucleosome structure	None	None	[78, 79]
	R131C	None	HFD	Interference with nucleosome structure	None	None	[78, 79]
H4	R3C	None	N-TT	Interference with residue symmetric demethylation and citrullination	None	None	[78, 79]
	D68Y/N/H	None	HFD	H2B-H4 interaction	None	None	[78, 79]

C-TT C-terminal tail, N-TT N-terminal tail, HFD histone fold domain

Interestingly, replication-independent histone variant H3.3 has attracted special attention since the two genes encoding this kind of histone are the ones in which specific amino acidic transitions are found at higher frequencies and, most importantly, mutations have been biologically characterized and strikingly associated with specific tumor types [78, 79, 112].

### K27M

The importance of histone H3 K27M mutation is underlined by the fact that it is the only histone mutation recognized by the World Health Organization (WHO) as a marker for tumor classification [113]. This oncohistone was first found in about 30% of pediatric high-grade glioma (pHGG) affecting the thalamus, the basal ganglia and the spinal cord [76, 77, 94]. K27M mutation was found primarily in H3F3A (about 75%), one of the two genes encoding the replication-independent H3.3, less frequently in the replication-dependent H3.1 (about 25%, mainly HIST1H3B and, to a lesser extent, HIST1H3C) and rarely in H3.2 (HIST2H3C) [76, 77, 114]. In 2016, the observation that this kind of oncohistone is present in 80% of patients affected by an aggressive form of pHGG named diffuse intrinsic pontine glioma (DIPG), led the WHO to classify K27M tumors as a separate subtype (diffuse midline glioma, H3K27M) [115].

Further studies indicate that this kind of oncohistone is frequently found also in adult cancers, such as glioma, acute myeloid leukemia and melanoma [78, 85, 95]. In many cases, H3.3K27M mutation was associated with a more aggressive phenotype [96]. In DIPGs, K27M mutations have been associated with mutations in TP53, PDGFRA, ACVR1 and BCOR [112].

**Fig. 3:**
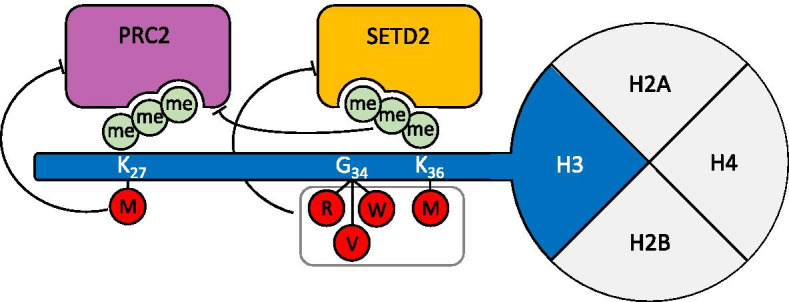
Proposed mechanisms of the main histone H3 mutations. H3K27M leads to a loss of H3K27me3 and H3K27me2 by acting as a dominant-negative inhibitor of PRC2, the complex responsible for H3K27 methylation. H3K36M oncohistone binds and dominantly inhibits the activity of SETD2, the histone methyltransferase responsible for H3K36 methylation. Methylation of H3K36 is known to antagonize the function of PRC2. H3G34 mutants block SETD2 binding, thus reducing its activity on H3K36 methylation. Mutations are indicated in red circles; methyl groups are shown as green circles

K27M mutation is associated with reduced H3K27me3 and H3K27me2 levels, increased H3K27ac and DNA hypomethylation [97–101]. Different studies demonstrate that this mutation leads to a loss of H3K27me3 and H3K27me2, and consequently to a loss of gene silencing, by acting as a dominant-negative inhibitor of the EZH2 writer subunit of PRC2, the complex responsible for H3K27 methylation (Fig. 3). The mutant protein induces this epigenetic perturbation even when incorporated at very low levels (3–17% of the total H3 population) [97–100]. A recent work shows that knockdown of this mutation in DIPG xenografts restores K27M-dependent loss of H3K27me3 and delays tumor growth. Interestingly, K27M-mediated loss of H3K27me3 seems to directly regulate a subset of differentiation genes by releasing poised (bivalent) promoters, possibly contributing to tumor phenotype and growth [102].

The dominant effect of H3K27 mutations on methylation seems to be specific to K27M (and to a lesser extent K27I). Similar effects were observed when other known methylated lysine residues of histone H3, such as H3K9 and H3K36, undergo K-to-M transitions, suggesting this kind of mutation as a mechanism to alter epigenetic states in cancer and other pathologies [97].

Different studies addressed the question of how K27M mutations exert dominant-negative activity, leading to the formulation of the Tethering and Sequestration model in which the ability of H3K27M to stabilize the binding of PRC2, and sequester it preventing further deposition of H3K27me, is considered the cause of the decrease in H3K27me [97–100]. However, although several studies support this model, many others contradict it, suggesting that the mechanism underlying H3K27M contribution to disease could be more complex than believed [103–108].

### K36M

Soon after the report of H3K27M in DIPG, K36M mutation was discovered in 73 of 77 cases of chondroblastoma (95%). Interestingly, 90% of the mutations were found in H3F3B, which is the other gene that codes for the variant histone H3.3. The mutation seems tumor type-specific as it was rarely found for example in bone cancers [84]. In addition, this mutation was found to be the only recurrent mutation in chondroblastoma, which was also scarcely associated with other genomic alterations. These observations indicate that H3K36M may play a major role in these tumors. H3K36M mutations at lower frequencies have also been reported in pediatric soft tissue sarcoma [88], head and neck squamous cell carcinoma [78, 89, 116], melanoma, bladder and colorectal cancer [78] although in these cases they preferentially target histone H3.1 rather than H3.3.

Immunocompromised mice subcutaneously injected with mesenchymal progenitor cells (MPCs) that express H3.3K36M mutant develop undifferentiated sarcomas [88]. T/C28a human chondrocyte progenitor cell line expressing H3.3K36M shows enhanced colony formation, less sensitivity to apoptotic stimuli and impaired homologous recombination [109]. These findings further support the involvement of H3.3K36M mutant in the oncogenic process.

H3.3K36M exerts dominant-negative effects on H3K36me2/3. Similarly to what already described for K27M, there is evidence supporting the enhanced ability of K36M oncohistone to bind and to dominantly inhibit the activity of SETD2, the histone methyltransferase responsible for H3K36 methylation (Fig. 3) [88, 109, 110]. Methylation of H3K36 has long been known to antagonize the function of the H3K27 methyltransferase EZH2 (Fig. 3). Thus, K36M mutation has been suggested to exert its oncogenic potential also by interfering with H3K27 methylation, although results from studies aimed to address this point are controversial [88, 109].

### G34 mutations

Another residue of histone H3 that attracted special attention in last few years is G34. As already documented for K27M and K36M, mutations of this residue normally target histone variant H3.3 and have been associated with specific cancer types affecting children and young adults [76, 84, 94]. However, differently from what was observed for the other two mutations, several cancer relevant transitions have been reported for G34. G34R and G34V have been described to be the more frequent [78]. Interestingly, such mutations targeting H3F3A gene have been found in approximately 14–15% of cortical pHGGs, with G34R being much more represented than G34V. Respect to K27M mutations, which are found in children and in tumors affecting the midline (thalamus, cerebellum, spine and pons), G34R/V mutations occur mainly in young adults and exclusively in tumors of the cerebral cortex [14, 76, 94]. The selective occurrence of K27M and G34R/V mutations in specific brain regions, as well as their prevalence in individuals of specific ages, suggests an exclusive developmental window and cell type that allow peculiar mutations in the young individual. In line with this hypothesis, H3.3G34R/V gliomas show early neuroprogenitor and interneuron-like transcriptomic signatures, while hindbrain and later neuroprogenitor signatures were found in H3.3K27M tumors [115]. G34R/V mutations were found to be associated with ATRX/DAXX mutations in pHGG [76].

Although not included among the most frequent histone mutations in cancer [78, 79], G34W and, to a lesser extent, G34L were found in H3F3A in about 90% of giant cell tumors of bone (GCTB), a locally aggressive benign tumor affecting young adults associated with extensive bone destruction [84].

G34W mutations of histone H3.3 have been observed also in a cancer syndrome involving pheochromocytomas and paragangliomas (PPGL) and GCTB, where the mutation is thought to arise postzygotically [117]. GCTB primary cells expressing G34W show increased colony formation, infiltration and proliferation, and G34W knock-in osteosarcoma MG63 cells recapitulate the same behavior [118]. In a recent study, G34W mutant GCTB primary cells showed impaired osteogenic differentiation associated with massive epigenetic alterations on DNA methylation, chromatin accessibility and histone modification affecting mainly heterochromatic and bivalent regions [111].

Structural studies suggest that G34 mutants may block SETD2 binding, thus reducing its activity on H3K36 methylation (Fig. 3) [119, 120]. However, unlike K36M, G34R/V/W mutants were found to decrease H3K36 methylation in cis (on the same histone H3) but not in trans, demonstrating the absence of dominant effects in contrast with their dominant-negative effects on cellular biology [97, 110, 121]. Histone modifications indeed occur only in sites of G34 mutant deposition, involving some reduction of H3K36me3 and a significant increase in H3K27me3 [121], similarly to what has been observed, at a global level, in K36M expressing cells [88]. HEK293 cells expressing H3.3G34R/V/D show similar in cis activity, with a significant decrease in both H3K36me2 and H3K36me [120]. Interestingly, H3.3G34R/V/D mutants showed reduced interaction with mismatch repair protein MutSα and a mutator phenotype similar to that of MMR-defective cells [120]. GBM specimens harboring G34 mutations show specific pattern of DNA methylation associated with global DNA hypomethylation, particularly at subtelomeric regions [94].

## Histone H2B mutations

Similarly to histone H3 mutations, those targeting H2B have been recently found to be among the most frequent histone mutations in tumors [78, 79]. However, unlike the other histones in which mutations occur at both the histone tail and the globular domain, mutated H2B residues lie mainly in the globular domain and, in most cases, are not sites of known PTMs [78, 79]. Nacev and colleagues indeed showed that **E76** and **E113** of histone H2B are among the 5 most commonly mutated histone residues, while Bennet and colleagues identified E76 as the most mutated histone residue in cancer (Fig. 2, Table 6) [78, 79]. This observation suggests that the structural role of histone H2B may be more important than that in chromatin regulation mediated by PTMs.

The most extensively studied transition of H2B is E76K. This transition, which is prevalently found in bladder and head and neck cancers targeting preferentially some genes of the Hist1 cluster (H2BC, H2BD, H2BF, H2BH and H2BI) [78, 79], is sufficient to distort the interface between H2B and H4 inducing nucleosome instability [90]. This effect occurs even if small amounts of the mutated histone are incorporated, suggesting dominant-negative abilities. Furthermore, E76K contributes to destabilize the nucleosome facilitating the dissociation of H2A/H2B dimers. The potential oncogenic activity of H2BE76K is suggested by the enhanced colony formation ability of cells expressing the mutated histone [90]. Expression of H2BE76K in the normal mammary epithelial cell line MCF10A enhances cellular proliferation and cooperates with mutant PIK3CA to promote colony formation, causing an important deregulation of gene expression accompanied by significant changes in chromatin structure [79].

As already stated, E113 is the second most mutated residue of histone H2B in cancer and one of the most mutated among all histone residues [78]. E113 mutations normally lead to E113K/Q transitions and seem to not involve specific H2B genes. Interestingly, this mutation targets a nucleosome anchoring point commonly referred to as “acidic patch,” a negatively charged groove formed by six H2A and two H2B residues at the H2A/H2B dimer surface [122]. Acidic patch mutations are described as promoting cellular transformation by interfering with several essential biological processes including chromatin condensation and folding, nucleosome remodeling, cell division, transcriptional silencing, and DNA damage repair [122].

Other H2B residues found to be frequently mutated in cancer are **F70** and **E71** [98, 99]. F70 mutations target mainly Hist2 cluster genes (in particular HIST2H2BE), while E71 mutations prevalently Hist1 cluster genes [79]. These residues are closed to the aforementioned E76 residue and are located in the same H2B-H4 interface. Interestingly, E71 of H2B and K91 of H4 form a salt bridge which is known to be disrupted by acetylation and ubiquitylation of H4 K91, affecting chromatin assembly and DNA damage repair [123, 124]. It is possible that E71 mutations could have similar consequences.

The only frequently mutated PTMs-targeted H2B residue in cancer is **E2**, that is among the 3–4 most mutated H2B residues [78, 79], with the most frequent transitions being E2Q and E2K. H2BE2 is a known site of ADP-ribosylation [125], although its regulatory role has never been deepened. Nevertheless, several studies have shown that ADP ribosylation of nucleosomes is associated with increased chromatin relaxation and that this modification may play an important role in the regulation of DNA repair, cell cycle progression and replication [126]. Other less frequent mutations affect also adjacent residues, such as P1, P3 and A4. Taken together, these observations suggest that E2 and its PTMs may play an important, still not elucidated, role in chromatin regulation and cancer.

H2B**G53** was identified as a frequent target of mutation in cancer [79], and a recent additional analysis of The Cancer Genome Atlas database showed the significant presence (4.5%) of G53D transition in pancreatic ductal adenocarcinoma (PDAC). G53D mutants increase the gap closure ability and transwell migration of cells, associated with an increased ability of RNA polymerase II to pass through the mutated histone. These observations suggest a possible effect of this mutant on chromatin relaxation and in the oncogenic process [92].

Other mutations of histone H2B were found in specific cancer types, such as the one producing **G27**A, **E36**G and **M63**K transitions in carcinosarcomas of the female genital tract [91] and the one targeting **S37** and **Y38** in follicular lymphomas [93]. While, on the one hand, the potential oncogenic effect of carcinosarcomas transitions was associated with structural perturbations of histone-histone and DNA–histone interactions, the residues targeted by follicular lymphoma mutations, which are phosphorylated in response to stress and during cell division, suggest an effect mediated by the interference with histone PTMs.

## Histone H2A mutations

Histone H2A mutations are not among the most frequent histone mutations in cancer [78, 79]. However, some quite frequent mutations affect also the genes encoding this histone, targeting both its terminal and its globular domain (Fig. 2, Table 6) [78, 79].

H2A mutations are commonly found in bladder cancer, endometrial carcinomas and head and neck carcinomas, although they have been described also in carcinomas of the female tract [79, 91]. Recent works show that the most frequently mutated residue of histone H2A is **E121**, with the most represented transitions being E121Q, E121K and, to a lesser extent, E121D [78, 79]. Most of these mutations target genes of the Hist1 cluster [79], suggesting that they may be dependent on genomic position. Interestingly, this residue lies in histone H2A *C*-terminal tail; histone H2A indeed is unique in having a protruding tail at both the *N*- and *C* termini. Footprinting and cross-linking experiments have shown that the *C*-terminal tail of histone H2A contributes to building the higher-order structures of chromatin, participating in the interaction of H2A-H2B dimer with H3–H4 dimer [127] and of H2A with linker DNA [128]. The truncation of histone H2A's C-terminal tail between the E121 and S122 has been linked to nickel(II)-induced carcinogenesis [129]. Although these evidences indicate a possible involvement of E121 mutations in perturbing higher-order structures of chromatin, the role of such mutations in cancer must still be clarified.

Frequent mutations target also **R29** of histone H2A producing in most cases R29Q transitions. Given that this arginine residue is subjected to methylation by PRMT6 methyltransferase and that H2AR29me2 has been associated with transcriptional repression [130], it is reasonable to assume that this mutation may exert oncogenic properties through the loss of H2AR29me2 and the consequent alteration of gene expression.

Interestingly, two adjacent lysine residues in the globular domain of histone H2A, **K74** and **K75**, show frequent lysine-to-asparagine mutations. This is one of the few cases in which lysine is substituted by asparagine since, as already described in previous paragraphs, it normally undergoes K-to-M or K-to-I transitions. Whether the frequency of K-to-N mutagenesis indicates a different functional implication compared to K-to-M/I mutations remains to be determined. Crystal structure studies indicate that these lysine residues are close to T80 of histone H3 and that they may participate in H2A-H3 [131] and H2A-DNA interactions [132].

Other mutations have been identified in carcinomas of the female tract such as **R4**H and **K16**T, which target known post-translational modified residues, and **E57**Q, which is involved in the maintenance of the structural integrity of the H2A fold [91].

The possible role of H2A.Z.1 mutations in cancer was also investigated. This prominent H2A variant indeed plays a role in important cellular processes, such as transcription, DNA repair and genomic stability. Interrogation of the cBioPortal identified high rates of R80C transition, and stability studies showed that this mutation produces less stable nucleosomes [90].

However, since no functional studies have been performed to date, a role of mutations targeting histone H2A and its variants in the oncogenic process can be only assumed analyzing the mutations frequency data from cancer genomic datasets.

## Histone H4 mutations

The only link between mutant histone H4 and cancer comes from the recent analysis of cBioPortal data [78, 79]. Interestingly, recent evidences suggest that alterations of genes expressing this histone can cause developmental syndrome [133, 134] and impact important cellular functions, such as nucleotide excision repair [135] and chromatin compaction [136].

Histone H4 genes are not among the most frequent mutated histone genes in tumors. Nevertheless, even in this case the most frequent H4 mutations encompass both the N-terminal domain and the globular domain (Fig. 2, Table 6).

**R3** was identified as the most frequent mutated residue of histone H4, with R3C being the most common mutation and with a balanced distribution across the different H4 genes [78, 79]. Interestingly, H4R3 PTMs, such as symmetric demethylation and citrullination, have been liked to transcriptional repression and response to DNA damage and their dysregulation was associated with cancer [137–140].

However, the distribution of recurrent histone H4 mutations suggests that the oncogenic potential of H4 mutants may lie on the disruption of chromatin folding and DNA packaging into nucleosomes rather than the alteration of histone PTMs [78, 79]. For example, the so-called switch-independent (Sin^−^) mutations, which cluster in the globular domains of histone H3 and histone H4 (residues 43–45), are known to abrogate the need for the SWI/SNF remodeling complex in regulating gene expression in yeast and to abolish higher-order chromatin folding [141]. Interestingly, **R45** of histone H4 was found to be frequently mutated in Nacev and colleagues study [79], while Bennet and colleagues work indicates gene H4B as the main target of such mutation [79].

Additionally, **D68** and **R92** were identified among the most mutated histone H4 residues in cancer patients. Interestingly, these residues are known to play a pivotal role in the structural interactions between histone H2B and H4. These residues, indeed, establish hydrogen bonds with H2B, and their alteration is thought to contribute to nucleosomes instability [78].

In addition, histone H4 K16 and the positive charge region R17-R23 domain are involved in chromatin folding through their interactions with the surface of the H2A/H2B dimer and, in particular, with H2A acidic patch [136]. Even in this case, a residue of this domain (**R17**) was found to be frequently mutated [78, 79].

## Conclusions

In the last few years, there is a growing interest in the role of epigenetic regulators and chromatin alteration in cancer development. Recurrent mutations targeting epigenetic factors have been identified in several types of cancer and, in many cases, are thought to play a main role in cancer development and progression. Starting from 2012, the discovery of highly frequent mutations in histone H3 genes in pediatric cancers drew great attention to the relation between histone mutations and cancer. Since that discovery, a number of recurrent mutations have been identified in all histones and some of them have been functionally characterized in cellular and animal models. Histone H3 mutations, such as K27M, K36M and G34 mutants, are among the most studied and showed oncogenic properties both in vitro and in vivo. Other characterized histone mutations are histone H2B E76K and histone H3 E97K, that were found to increase colony formation and proliferation in cellular models, while histone H2B G53D and histone H2A E57Q were found to impact cellular migration in vitro (Table 6).

Contrary to what was expected, the accumulated knowledge revealed that recurrent mutations are not restricted to the histone tails, normally involved in chromatin regulation mediated by PTMs at specific residues, but that, on the contrary, they are mainly represented in the globular domain. This finding indicates the destabilization of nucleosome, DNA–nucleosome interactions and the disruption of higher-order chromatin structure as a major mechanism of the oncohistones biological activity. In line with this observation, several oncohistones were found to impact chromosomal stability in different models, suggesting that this could be a main mechanism of oncohistone-mediated tumorigenesis.

Interestingly, histones mutations target specific genes among many expressing the same histone type, but the reasons of such phenomenon are far to be fully elucidated. In some cases, it has been shown that genes coding the same histone type carry different codons for the same amino acid causing the same mutation to produce different residue transitions. In other cases, mutations target at higher rates genes of a specific cluster, suggesting that position may have a role. In most cases, it seems that no apparent explanation can be given, although it has been suggested that the targeting of specific genes might be due also to the differential expression of these genes in the cell of origin [14].

Histone mutants may exert dominant effects regardless of the presence of several not-mutated copies of their genes and with the mutant protein constituting only a small fraction of the histone proteins of the same family. A model in which the ability of the mutant to stabilize the binding of epigenetic factors, and sequester them preventing further modifications, has been proposed but is still debated.

Another peculiar feature of oncohistones is that specific recurrent amino acidic transitions are often associated with specific tumor types or, as documented for histone variant H3.3 in glioma, with peculiar tumors arising from specific anatomic locations and at specific ages. This selective occurrence suggests an exclusive developmental window and cell type that allow peculiar mutations in the young individual. This observation, together with others such as the association between developmental syndrome and histone H4 mutations, the perturbation of differentiation genes induced by K27M in DIPG and the reduced differentiation of G34W mutants in GCTB, indicates the interference with development and differentiation as a common theme of oncohistones.

Histone mutations, especially those affecting histone H3, are the most frequent in tumors such as DIPG, chondroblastoma and GCTB and in some cases, as for K36M in chondroblastoma, they represent the only recurrent mutation. This evidence suggests a role of these oncohistones as drivers of tumorigenesis in these tumors. However, in many cases mutations that have not been identified as recurrent in specific tumors were found to be frequent when analyzed across several cancer types. This kind of analysis is extremely relevant as it allowed to bring out the previously underestimated importance of many histone mutations. In fact, since histone mutations are distributed over several histone genes, they probably suffer from a reduced visibility in lists of individual genes from sequencing studies. However, if the data to date available demonstrate a main role of histone mutations as drivers in different pediatric cancers, this seems to be less true in adult cancers where their recurrence is lower, suggesting they may have a secondary role.

The demonstration that pHGGs present a high frequency of mutations in histone genes, associated with chromatin alterations, suggests that these tumors might be responsive to epigenetic therapies. Attempts focused on the inhibition of histone demethylases, increase in histone acetylation using histone deacetylase (HDAC) inhibitors and EZH2 inhibition provided good results against H3K27M expressing cells [105, 142–144], and several clinical trials across different tumors have started [15].

Although the promise of the field is exciting, further studies are necessary to address the many questions that remain still open, to elucidate the functional role of many histone mutations, as well as to evaluate the possible expansion of the application of histone mutants to cancer classification, prognosis and therapy.

## Data Availability

Not applicable.

## Abbreviations

DIPG: Diffuse intrinsic pontine glioma
EMT: Epithelial-mesenchymal transition
HDAC: Histone deacetylase
H-NS: Histone-like nucleoid-structuring protein
HU: Histone-like proteins
PDAC: Pancreatic ductal adenocarcinoma
pHGG: Pediatric high-grade glioma
pGBM: Pediatric glioblastoma
PTMs: Post-translational modifications
WHO: World Health Organization
